# Genome-wide bioinformatics analysis of Cellulose Synthase gene family in common bean (*Phaseolus vulgaris* L.*)* and the expression in the pod development

**DOI:** 10.1186/s12863-022-01026-0

**Published:** 2022-01-29

**Authors:** Xiaoqing Liu, Hongmei Zhang, Wei Zhang, Wenjing Xu, Songsong Li, Xin Chen, Huatao Chen

**Affiliations:** Jiangsu Academy of Agricultural Sciences, Jiangsu Key Laboratory for Horticultural Crop Genetic Improvement, Institute of Industrial Crops, Nanjing, 210014 China

**Keywords:** Cellulose synthase genes, Common bean, Phylogenetic relationships, Gene expression

## Abstract

**Background:**

*CesA* and *Cs*l gene families, which belong to the cellulose synthase gene superfamily, plays an important role in the biosynthesis of the plant cell wall. Although researchers have investigated this gene superfamily in several model plants, to date, no comprehensive analysis has been conducted in the common bean.

**Results:**

In this study, we identified 39 putative cellulose synthase genes from the common bean genome sequence. Then, we performed a bioinformatics analysis of this gene family involving sequence alignment, phylogenetic analysis, gene structure, collinearity analysis and chromosome location. We found all members possess a cellulose_synt domain. Phylogenetic analysis revealed that these cellulose synthase genes may be classified into five subfamilies, and that members in the same subfamily share conserved exon-intron distribution and motif compositions. Abundant and distinct *cis*-acting elements in the 2 k basepairs upstream regulatory regions indicate that the cellulose synthase gene family may plays a vital role in the growth and development of common bean. Moreover, the 39 cellulose synthase genes are distributed on 10 of the 11 chromosomes. Additionally expression analysis shows that all *CesA/Csl* genes selected are constitutively expressed in the pod development.

**Conclusions:**

This research reveals both the putative biochemical and physiological functions of cellulose synthase genes in common bean and implies the importance of studying non-model plants to understand the breadth and diversity of cellulose synthase genes.

**Supplementary Information:**

The online version contains supplementary material available at 10.1186/s12863-022-01026-0.

## Background

In plants, there exists a cellulose synthase superfamily including *CesA* (cellulose synthase) and *Csl* (cellulose synthase-like) gene family, both of which belong to the glycosyltransferase GT2 family and have a similar protein sequence structure. The encoded proteins all have glycosyltransferase activity and are key enzymes essential for cellulose and hemicellulose synthesis [1, 2], which are main components of the primary and secondary cell wall.

The cellulose synthase (*CesA*) gene was first identified from cotton fibers according to its sequence similarity with a bacterial *CesA* gene [3]. Subsequently, cellulose synthase genes were identified in Arabidopsis [4], rice [5], and maize [6], barley [7]. The CesA family contains a conserved motif (DDDQxxRW) and a zinc-finger domain [8]. In *Arabidopsis thaliana*, AtCesA1, AtCesA3, and AtCesA6 form a cellulose synthase complex and participate in the synthesis of the primary cell walls. Whereas, AtCesA4, AtCesA7, and AtCesA8 mediate in the synthesis of the secondary cell walls. It is generally accepted that despite the involvement of most CesA in the synthesis of the primary and secondary cell wall [9–11], AtCesA2, AtCesA5, AtCesA9 are considered homologous proteins of AtCesA6, and these proteins are functionally redundant with each other.

*Csl* (cellulose synthase-like) genes, which share a relatively high sequence similarity to *CesA* (cellulose synthase A) genes [12], is divided into 9 subfamilies, in which *CslA* and *CslC* are distantly related to the other families; *CslA*, *CslC*, *CslD* and *CslJ* subfamilies are ubiquitously present in all terrestrial plants [5], *CslF* and *CslH* are specific to monocots, *CslB* and *CslG* are thought to be unique to eudicots [13, 14]. There are many evidence supporting *CSl* gene family involving in the biosynthesis of cell wall polysaccharides. CslA catalyzes the synthesis of (1,4)-β-D-mannan [15–18], and CslC is involved in catalyzing the formation of the xyloglucan cytoskeleton [18–20]. Meanwhile, CslD also plays a vital role in xylan and galactoaldoglycan synthesis [21–24]. Additionally, CslF is a gene family unique to monocotyledons, and mediates the synthesis of β-(l,3;l,4)-D-glucan [25, 26]. However, at present, the biological functions of CslB, CslE, CslG and CsJ gene families remain unknown.

Not only a major source of protein and essential nutrients, common bean (*Phaseolus vulgaris* L.) is also an important crop to society and the global economy [27]. However, the CesA/Csl gene families in common bean have not yet been extensively explored. Molecular biology, genomics, and computational biology have transformed the field of biology, gene discovery and functional gene annotation in plant genome-wide data is a rapidly growing research area. Considering the critical role of CesA/Csl in both the integrity and function of plants, we present a comprehensive phylogenetic and functional bioinformatics analysis of the *CesA/Csl* gene family in common bean. Then, using quantitative real-time polymerase chain reaction (qRT-PCR) analysis on genes identified in our computational pipeline, we validate the main genes central for the development of legume pods. These findings shed new light on the relationship between *CesA/Csl* function and the development of common bean. Furthermore, this research presents a theoretical framework for gene cloning and expression in the future, with the application of genetically improving the common bean through breeding.

## Results

### Identification of cellulose synthase genes in common bean

In order to identify the cellulose synthase gene family of common bean, first, the Hidden Markov Model of 40 *Arabidopsis* cellulose synthase proteins was constructed, then the model was used as queries to search against the common bean protein databases with the BLASTP program at an e-value threshold of 10–10. Then, we searched for the cellulose synthase gene family of common bean using the constructed model and finally a total of 39 sequences can matched to *CesA/Csl* superfamily. 14 gene members contained a cellulose synthase domain (CS) and zinc finger structure (zf-UDP), 25 gene members only harbored a CS domain. The identified cellulose synthase proteins were named according to the order of their subfamilies and gene IDs.

These putative cellulose synthase genes in this analysis were predicted to range from 467 to 1374 amino acids in length and 53.34 kDa to 155.53 kDa in molecular weight. Furthermore, the protein isoelectric points (pIs) ranged from 5.62 to 9.05, the number of predicted TMHs ranged from 0 to 13. The subcellular localization of the putative cellulose synthase genes was predicated to be located in the membrane bound golgi and plasma membranes PHAVU_005G116500g, which were exist in extracellular (secreted) (Table 1).

**Table 1:** Classification and characterization of the putative Cellulose Synthase genes in *Phaseolus vulgaris*

Gene_name	Family	Chromosome.No	Amino acid no.	Molecular weight (Da)	Isoelectric points	Location	Number of predicted TMHs	Domain
PHAVU_001G211000g	CslD	1	1149	127,991.62	8.49	Membrane bound Golgi	6	CS (PF03552)
PHAVU_002G040200g	CslD	2	1144	127,998.61	7.06	Membrane bound Golgi	8	CS (PF03552)
PHAVU_002G136300g	CslD	2	1117	125,646.3	6.11	Membrane bound Golgi	8	CS (PF03552)
PHAVU_002G188600g	CesA	2	1034	117,583.67	8.18	Plasma membrane	8	zf-UDP (PF14569), CS (PF03552)
PHAVU_002G240200g	CesA	2	976	110,235.69	5.89	Plasma membrane	6	zf-UDP (PF14569), CS (PF03552)
PHAVU_002G268200g	CesA	2	1097	123,785.06	6.66	Plasma membrane	6	zf-UDP (PF14569), CS (PF03552)
PHAVU_003G023000g	CslD	3	992	111,961.41	8.65	Membrane bound Golgi	8	CS (PF03552)
PHAVU_003G154600g	CesA	3	1031	116,535.77	6.09	Plasma membrane	6	zf-UDP (PF14569), CS (PF03552)
PHAVU_003G290600g	CslG	3	734	84,006.68	7.23	Plasma membrane	7	CS (PF03552)
PHAVU_004G093300g	CesA	4	1089	123,074.89	6.76	Plasma membrane	8	zf-UDP (PF14569), CS (PF03552)
PHAVU_005G001000g	CslG	5	700	79,831.81	8.04	Plasma membrane	5	CS (PF03552)
PHAVU_005G010400g	CesA	5	1033	117,479.39	6.23	Plasma membrane	8	zf-UDP (PF14569), CS (PF03552)
PHAVU_005G022100g	CesA	5	1075	120,033.65	6.94	Plasma membrane	6	zf-UDP (PF14569), CS (PF03552)
PHAVU_005G116200g	CslB	5	743	84,181.59	8.38	Membrane bound Golgi	9	CS (PF03552)
PHAVU_005G1163000g	CslB	5	528	58,607.06	7.55	Plasma membrane	6	CS (PF03552)
PHAVU_005G1164000g	CslB	5	520	57,674.75	6.18	Plasma membrane	5	CS (PF03552)
PHAVU_005G116500g	CslB	5	1374	155,531.98	9	Extracellular (Secreted)	13	CS (PF03552)
PHAVU_005G116700g	CslB	5	750	85,099.39	7.5	Membrane bound Golgi	6	CS (PF03552)
PHAVU_006G058400g	CslE	6	738	84,196.75	8.22	Membrane bound Golgi	7	CS (PF03552)
PHAVU_006G0586001g	CslE	6	528	60,056.71	8.08	Membrane bound Golgi	6	CS (PF03552)
PHAVU_006G058700g	CslE	6	752	86,312.44	7.14	Membrane bound Golgi	7	CS (PF03552)
PHAVU_007G081700g	CesA	7	1093	123,205.98	6.39	Plasma membrane	6	zf-UDP (PF14569), CS (PF03552)
PHAVU_007G130400g	CslG	7	741	84,593.15	5.94	Plasma membrane	7	CS (PF03552)
PHAVU_007G190300g	CesA	7	884	100,646.12	6.26	Plasma membrane	8	CS (PF03552)
PHAVU_008G193000g	CslD	8	1128	125,931.82	5.89	Membrane bound Golgi	6	CS (PF03552)
PHAVU_008G279600g	CslE	8	748	85,229.48	8.68	Membrane bound Golgi	6	CS (PF03552)
PHAVU_008G279700g	CslE	8	1006	114,636.64	8.54	Membrane bound Golgi	10	CS (PF03552)
PHAVU_008G279800g	CslE	8	744	85,290.55	8.41	Membrane bound Golgi	7	CS (PF03552)
PHAVU_009G090100g	CesA	9	968	109,222.66	6.33	Plasma membrane	8	zf-UDP (PF14569), CS (PF03552)
PHAVU_009G094200g	CesA	9	1084	122,269.89	6.35	Plasma membrane	6	zf-UDP (PF14569), CS (PF03552)
PHAVU_009G205100g	CesA	9	1041	117,486.26	6.31	Plasma membrane	8	zf-UDP (PF14569), CS (PF03552)
PHAVU_009G205200g	CesA	9	974	109,369.83	5.97	Plasma membrane	6	zf-UDP (PF14569), CS (PF03552)
PHAVU_009G2260000g	CslG	9	505	57,280.95	9.05	Plasma membrane	5	CS (PF03552)
PHAVU_009G242700g	CesA	9	1048	118,882.58	7.95	Plasma membrane	8	zf-UDP (PF14569), CS (PF03552)
PHAVU_011G020100g	CslD	11	1148	128,959.97	7.04	Membrane bound Golgi	8	CS (PF03552)
PHAVU_011G101500g	CslB	11	746	84,596.22	8.42	Membrane bound Golgi	6	CS (PF03552)
PHAVU_011G211500g	CesA	11	1074	120,142.75	6.68	Plasma membrane	6	zf-UDP (PF14569), CS (PF03552)
PHAVU_L008300g	CslE	Scaffold_191	648	72,876.83	6.63	Membrane bound Golgi	5	CS (PF03552)
PHAVU_L009400g	CslB	Scaffold_243	467	53,343.3	5.62	Plasma membrane	2	CS (PF03552)

*CesA* Cellulose synthase A, *Csl* Cellulose synthase-like

### Phylogenetic analysis of cellulose synthase gene in common bean

**Fig. 1:**
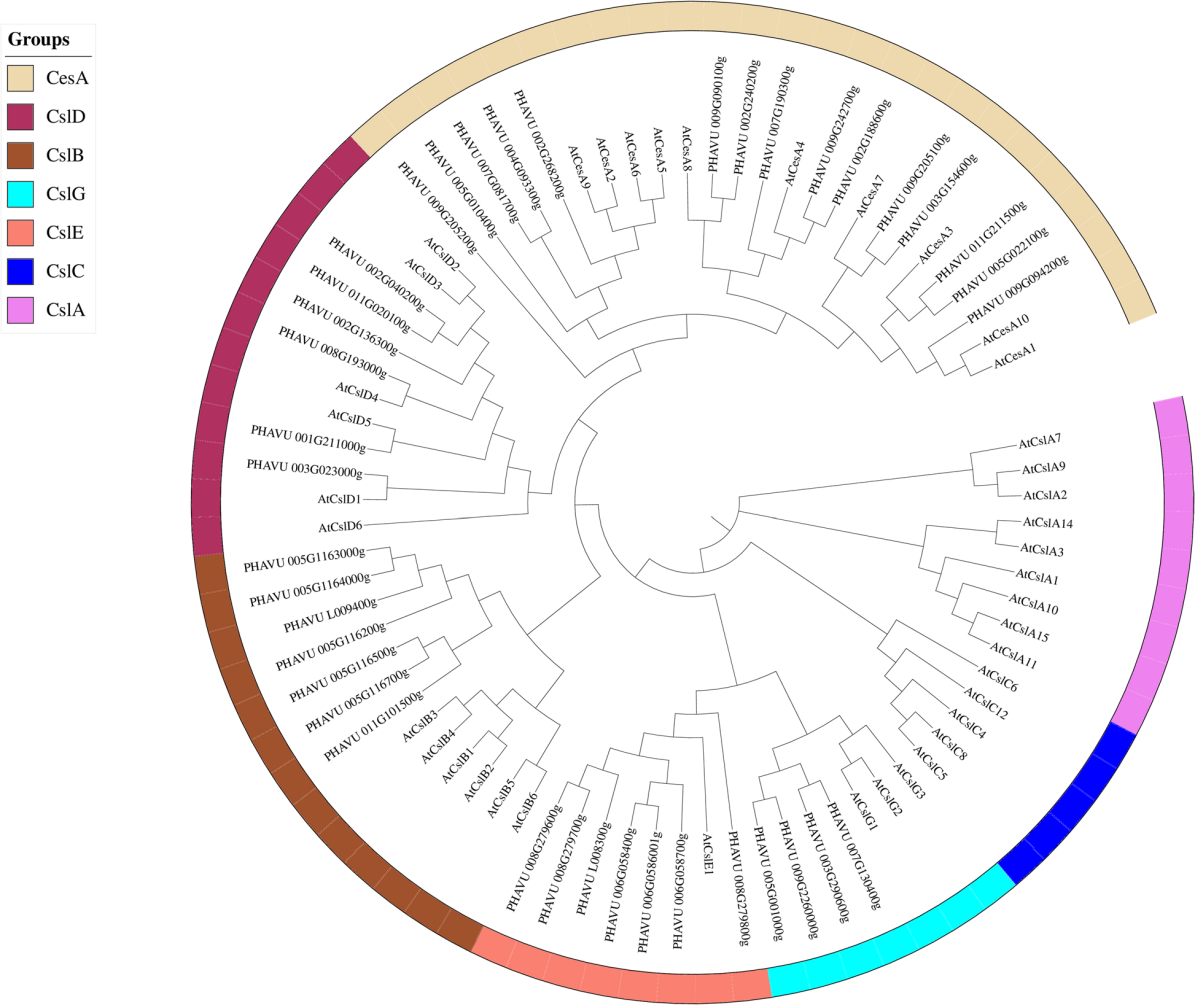
Phylogeny of putative cellulose synthase genes from *Arabidopsis thaliana* and *Phaseolus vulgaris*. Bootstrap values for 1000 replicates are indicated on each branch. CesA (Cellulose synthase A) family, Csl (Cellulose synthase-like protein) family

A phylogenetic analysis was used investigate the evolutionary relationships among cellulose synthase proteins. Constructed with cellulose synthase proteins from Arabidopsis and 39 from common bean, the phylogenetic analysis showed that 15 putative cellulose proteins from common bean belong to the CesA family, while the remaining 25 cellulose synthase proteins are members of the Csl (B, D, E, and G) family (Fig. 1). CslD is close to CesA, while CslG is distantly related to the other families.

### Gene structure analysis of cellulose synthase gene in common bean

**Fig. 2:**
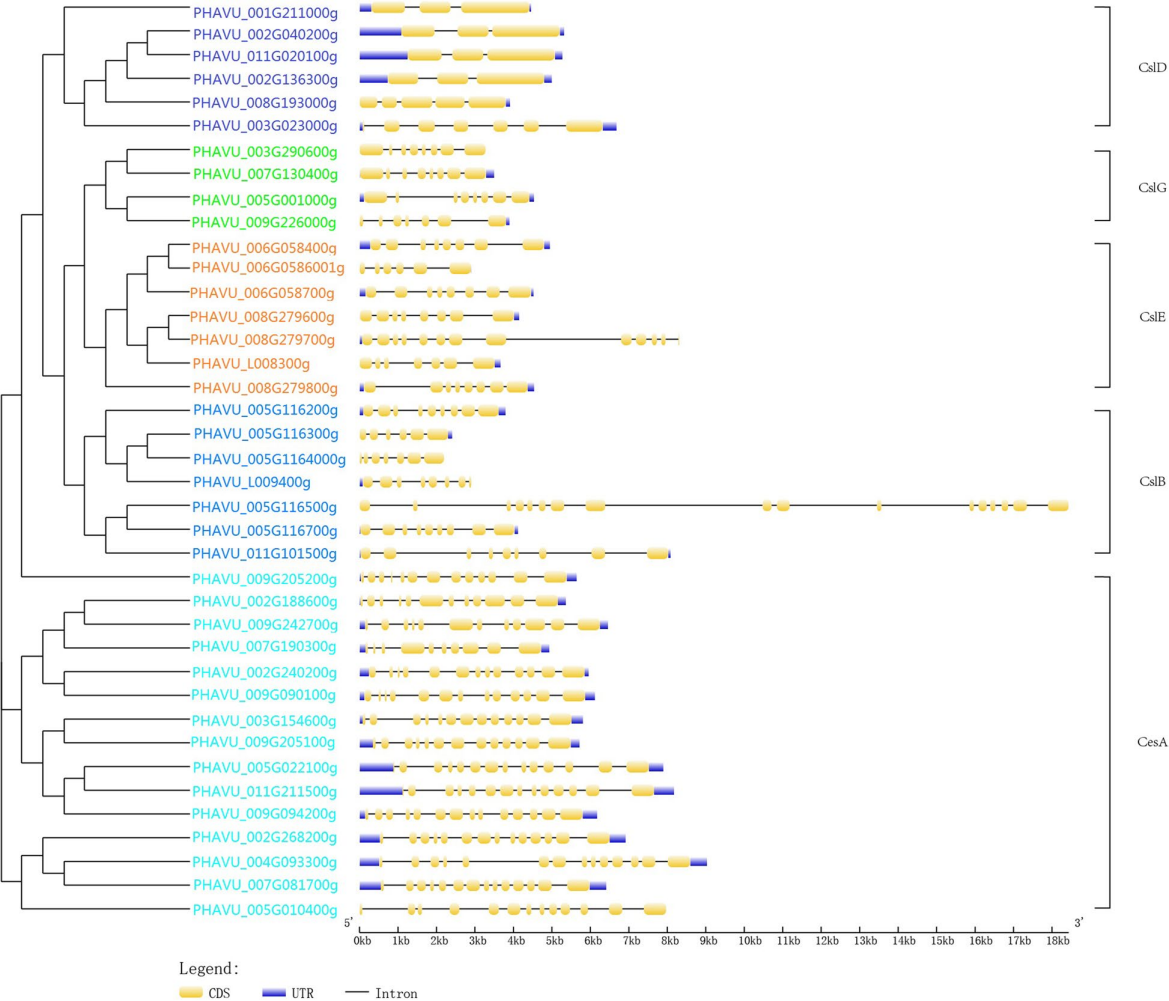
Gene structure of the cellulose synthase genes in *Phaseolus vulgaris*. Yellow and blue boxes represent exons and UTRs, respectively, black lines represent introns. The lengths of the exons, introns, and UTRs were drawn to scale

Exon-intron structures of each *CesA/Csl* gene were constructed through the sequence alignment of their corresponding genomic DNA. Based on the phylogenetic analysis, putative *CesA/Csl* genes’ exon/intron structures in common bean were organized into five subgroups (Fig. 2). *CesA/Csl* genes in the same subgroup had conserved exon/intron structures, while genes in different groups exhibited distinct gene structures. And we found that *CesA* gene members had the most introns, while the *CslD* gene members had the fewest number of introns.

### Conserved motif domains of CesA/Csl gene in common bean

**Fig. 3:**
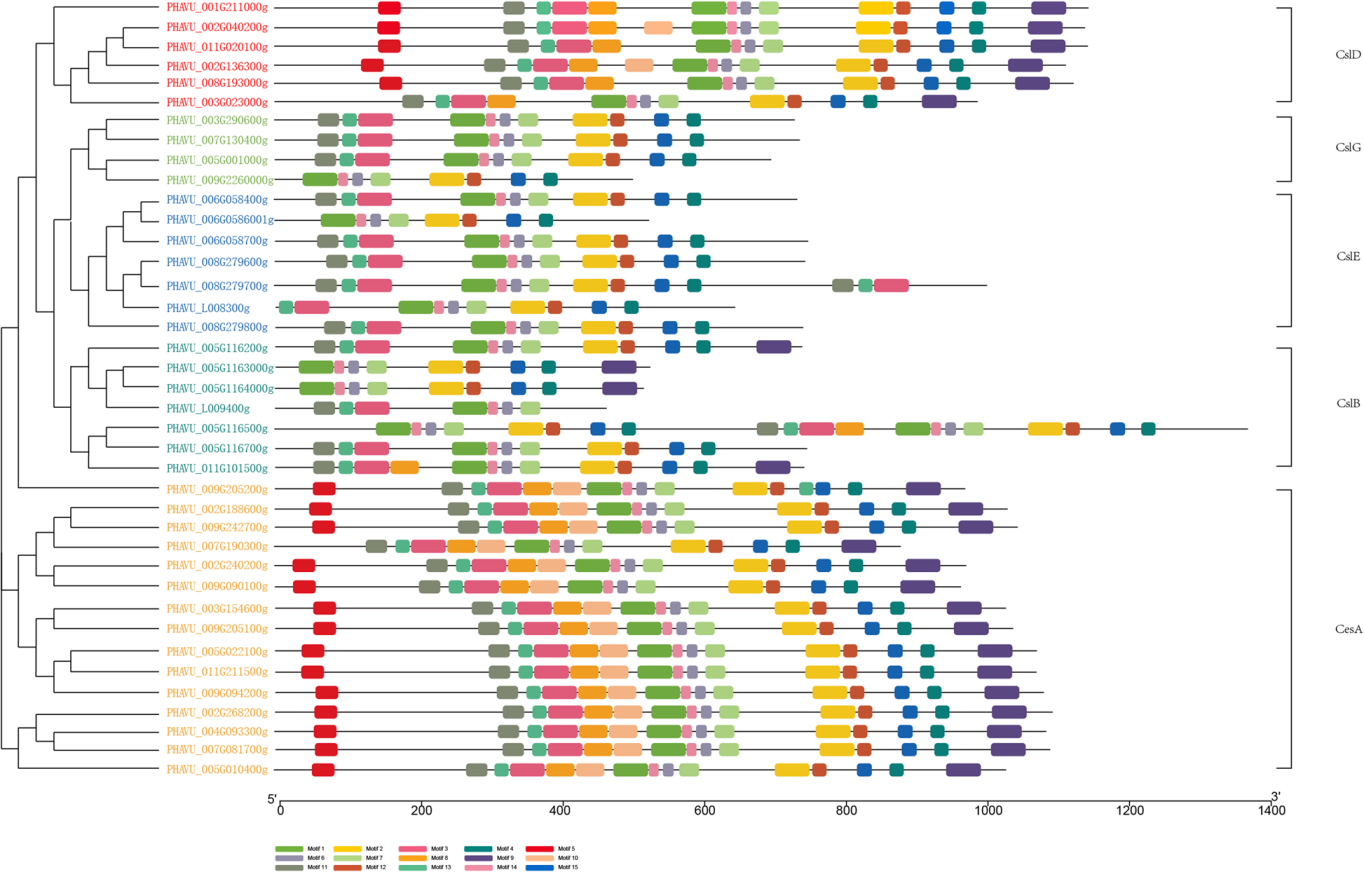
Analysis of the conserved motifs of putative cellulose synthase genes in *Phaseolus vulgaris*. Boxes with different colors represent different motifs and correspond to the location of each cellulose synthase protein. Detailed information on the 15 motifs is provided in Supplementary Material File 1

To evaluate the structural diversity of cellulose synthase proteins, we used the online program MEME (http://meme.sdsc.edu/meme/cgi-bin/meme.cgi) to search for conserved motifs in putative cellulose synthase protein sequences (Fig. 3). We identified 15 conserved motifs, and found that members in the same subfamily shared similar conserved motifs (Fig. 3). In addition, members in the CesA and CslD groups contained more motifs than members in CslB,, CslE and CslG groups (except PHAVU_005G116500).

### Promoter regions analysis of CesA/Csl genes

**Fig. 4:**
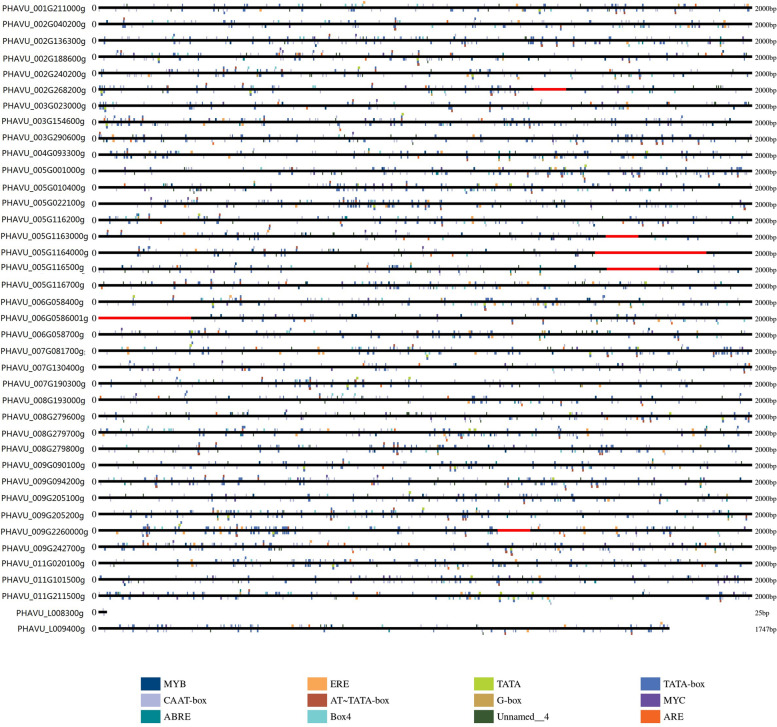
Prediction of *cis*-responsive elements in the 2 k upstream regulatory regions of the putative cellulose synthase genes. Different *cis*-responsive elements are represented by different colored boxes. Detailed information of promoter elements is illustrated in Supplementary Material File 2

To identify the cis-elements in the promoters of *CesA/Csl* genes in common bean, the 2000 bp basepairs upstream of the start codon of each gene were analysed using PlantCARE online (http://bioinformatics.psb.ugent.be/webtools/plantcare/html/). The results showed that abundant cis-elements were present in the promoters of *CesA/Csl* genes (except PHAVU_L008300). CAAT-box and TATA-box were the most abundant elements. TATA-box, a core promoter element, which located in about 30 bp upstream of the transcription start site, while CAAT-box is a common *cis*-acting element in promoter and enhancer regions. Also present were MYB transcription factor binding sites (TAACCA), light response element Box 4, and stress response elements, including MYC (in response to drought stress) and ARE (related to anaerobic stress). In addition, hormone response elements, ERE and ABRE, were observed, which respond respectively to ethylene and abscisic acid. The *cis*-acting elements had diverse functions and abundant types, indicating that the cellulose synthase gene family may plays an important role in the growth and development of common bean (Fig. 4, Supplementary Material File 2).

CAAT-box: a common *cis*-acting element in promoter and enhancer regions; TATA: a core promoter element located about 30 bp upstream of the transcription start site; MYB: MYB recognition site; G-box: *cis*-acting regulatory element involved in light responsiveness; TATA-box: a sequence of DNA, consisting of nucleobases TATAAA, located in the promoter region about 25 base pairs before the site of transcription; MYC: *cis*-acting element involved in drought and abscisic acid responsiveness; Box4: conserved DNA module involved in light responsiveness; AT-TATA-box:; ERE: ethylene-responsive element; ABRE: *cis*-acting element involved in abscisic acid responsiveness; ARE: *cis*-acting regulatory element essential for the anaerobic induction.

### Chromosome location of CesA/Csl genes in common bean

**Fig. 5:**
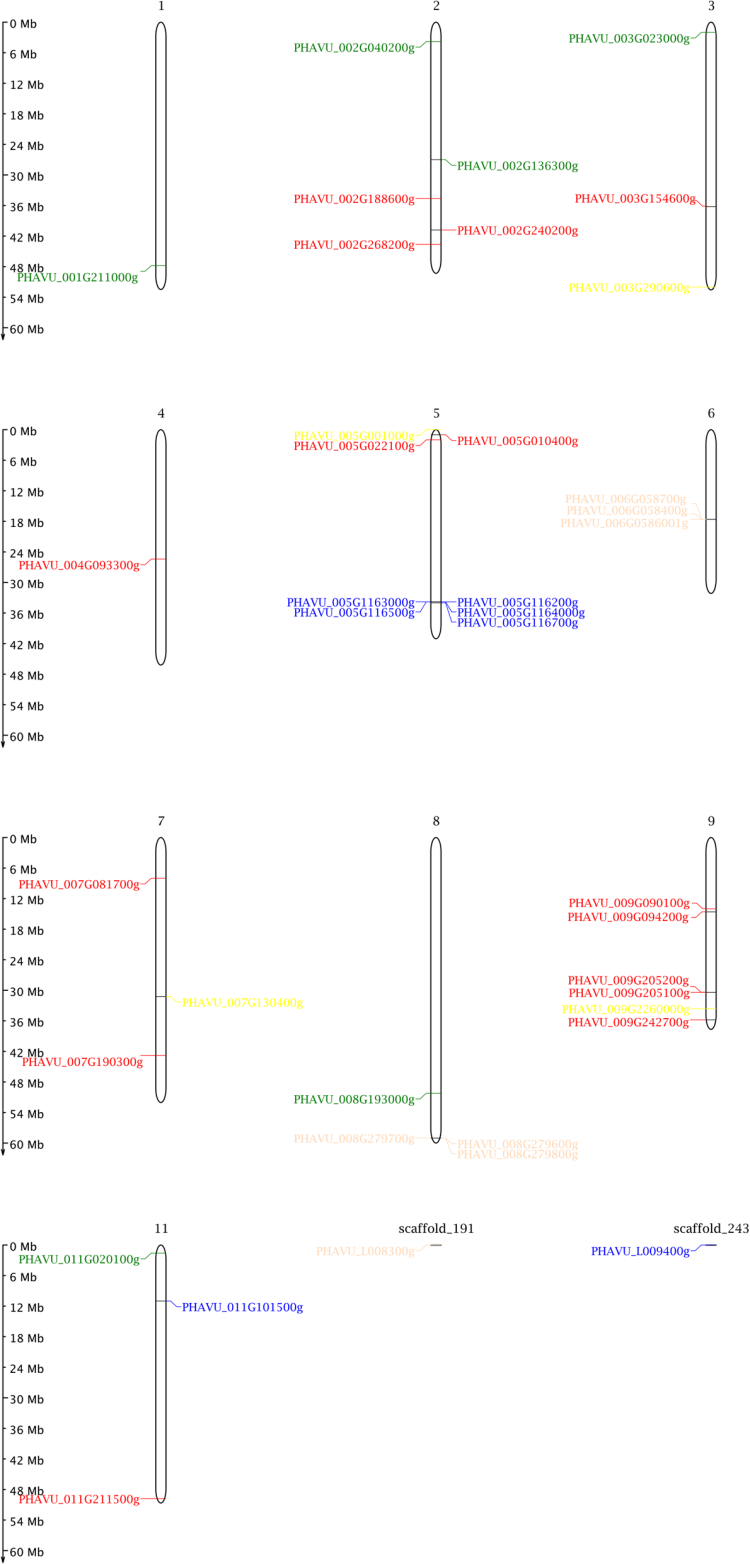
Chromosome map of the cellulose synthase gene family. Gene name color corresponds to gene family/sub family. i.e. green (CslD), red (CesA), yellow (CslG), blue (CslB), light pink (CslE). A scale on the left represents the length of chromosome in megabases (Mb)

Then, we investigated the chromosome distribution of the 39 cellulose synthase genes using the physical locations of the sequences on the chromsomes of common bean. As demonstrated in the location image, 39 *CesA/Csl* gene members are distributed on 10 chromosomes, and no genes mapping to chromosome 10 (Fig. 5). Chromosome 5 contained the largest number of cellulose synthase gene members (eight), followed successively by chromosome 9 (six), chromosome 2 (five), and Chromosomes 3, 6, 7, and 11 (three). Besides, we found 3 tandem duplication sets: PHAVU_005G1163000g (CslB)/ PHAVU_005G116500g (CslB)/ PHAVU_005G116200g (CslB)/ PHAVU_005G1164000g(CslB)/ PHAVU_005G116700g(CslB), PHAVU_006G058400g(CslE)/ PHAVU_006G058700g(CslE), PHAVU_008G279700g(CslE)/ PHAVU_008G279600g(CslE)/ PHAVU_008G279800g(CslE).

**Fig. 6:**
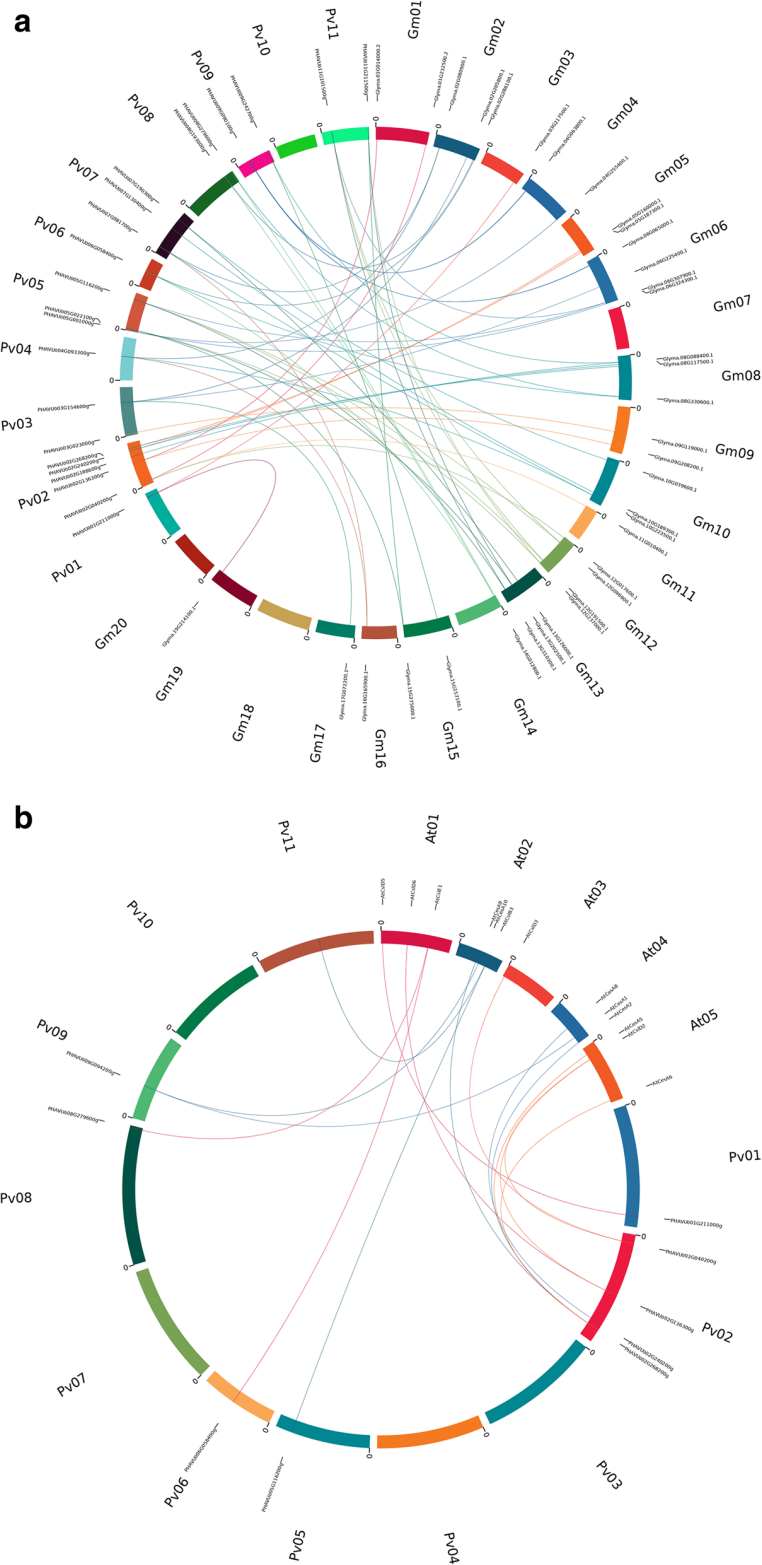
Synteny analysis of cellulose synthase gene family in *Phaseolus vulgaris* with the cellulose synthase gene family in *Glycine max* (**A**) and *Arabidopsis thaliana* (**B**). Synteny map was constructed by using online tool Circoletto: visualizing sequence similarity with Circos. Color variations represent the extent of similarity and homology between genes based on bit score. Detailed information of the sequences is provided in Supplementary Material File 3, 4, 5

The comparative synteny relationship map of *Phaseolus vulgaris* revealed a high degree of similarity with *Glycine max* (Fig. 6A) and a low degree of similarity with *Arabidopsis thaliana (*Fig. 6B).

### Expression Profiles of CesA/Csl genes in common bean pod development

**Fig. 7:**
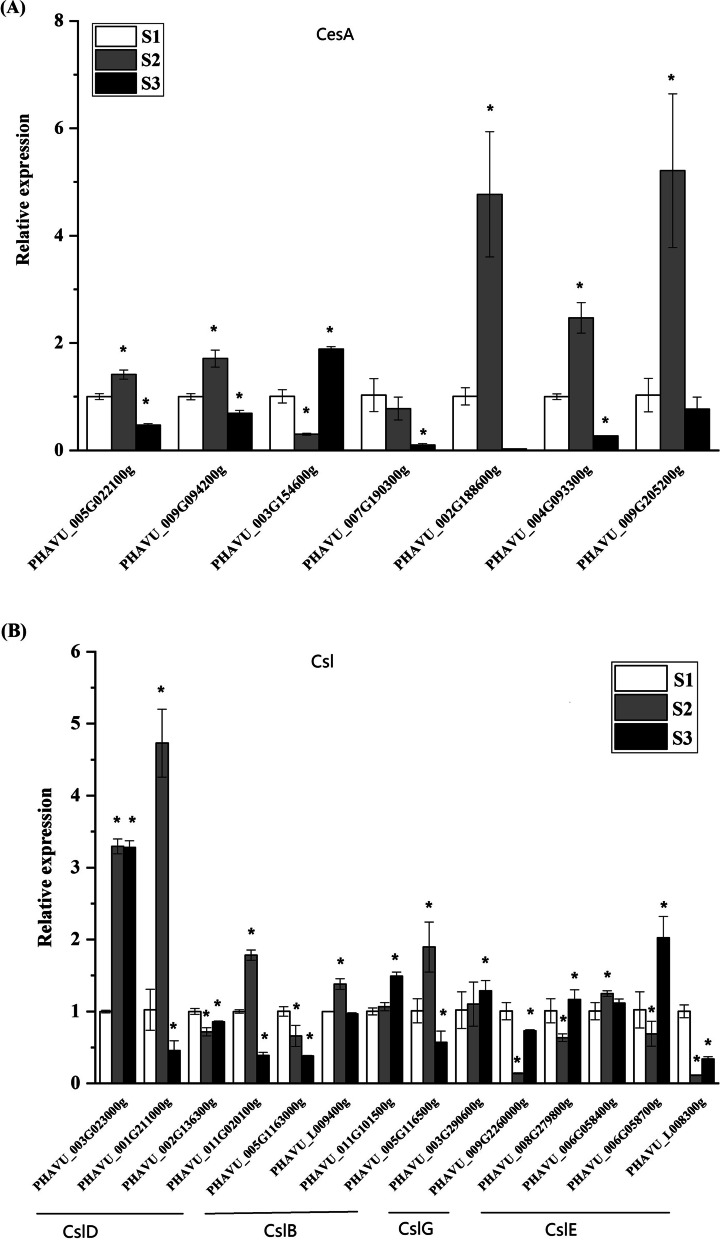
Expression analysis of 6 *CesA* (**A**), 4 *CslD*, 4 *CslB*, 2 *CslG*, 4 *CslE*, (**B**) genes at three seed developmental stages of *Phaseolus vulgaris*. To visualize the relative expression levels data, S1 stage was normalized as “1”, and data are means ± SD calculated from three biological replicates. * indicate significant differences in comparison with the control at *p* < 0.05

To investigate the functions of *CesA/Csl* genes in common bean pod development, we used RT-qPCR with gene-specific primers (Table 2) to analyze the expression levels of *CesA/Csl* genes at three distinct pod developmental stages. A total of 21 *CesA/Csl* genes included in this analysis were selected based on results from the sequence alignments, phylogenetic analysis, and gene structure analysis. All *CesA/Csl* genes (seven CesA, four CslD, four CslB, two CslG and four CslE) were expressed at all three stages of pod development, suggesting their important roles in the development of the pod in common bean (Fig. 7A, Fig. 7B).

**Table 2:** Gene-specific DNA primers for qPCR

Gene_name	Primer sequence (5′ –3′)
*PHAVU_005G022100g*	F-TGAGGTGGAGTGGTGTTGGA; R-GGAGGGATGAGGAGGGTTGT
*PHAVU_009G094200g*	F-AAGAAGAGGGCGATGAACAGA; R-TCCATGAAAGTGGCAGCAATA
*PHAVU_003G154600g*	F-CTGATGACGGAGCTTCAATGTG; R-TGCACGAGGTTCGATAGAAAA
*PHAVU_007G190300g*	F-GTGGGAATTGTGGCTGGAA; R-TGCTGGACCTGTCTGCTTGG
*PHAVU_004G093300g*	F-TCCTTGTTGATCCCTCCCTT; R-CCTGTTTACCCATGACACCCT
*PHAVU_009G205200g*	F-AGGCAGAACAGAACACCAACTC; R-TTCCACATTGTTTGGCATCAG
*PHAVU_003G023000g*	F-GGCAGGAGGATCAGAACACTT; R-CGGACAACCACCAACAACC
*PHAVU_001G211000g*	F-GACGAGGAAGCAATGAAAGGC; R-ATGGAAGGCAGAGGCAGAGG
*PHAVU_002G136300g*	F-CCCTTGTCATCCTTGCTGTTT; R-TCAATGCCAGCCATCACCT
*PHAVU_011G020100g*	F-ATTCGGTTGGTTGTCCTGGTA; R-CAGTAGGATTGTTGGGACTTCG
*PHAVU_005G1163000g*	F-GGAAATGGGAGTGGAATAGGA; R-GATGACATGCAAATGGTGGTTA
*PHAVU_L009400g*	F-CCGTAACTCACCCAGATCGTC; R-GGCTTCCACAAGGGCATAGA
*PHAVU_011G101500g*	F-CAATTTCTTGCCACAGGAGC; R-AACCAGCATTCATGGGTGTTAT
*PHAVU_005G116500g*	F-ATCAAGACGGGACGTGACAGA; R-TGACATCAAGCGGGTTATCG
*PHAVU_003G290600g*	F-TTGCCGGAGGATCACAAAC; R-CCATGCAGGGTCAAAGGAGA
*PHAVU_009G2260000g*	F-TGCCAATGGCGGTTTATG; R-GCAACATCTTTGGAGGTTTCAG
*PHAVU_008G279800g*	F-TTGCACCTATGAGGAGGGC; R-CCCATAAATGAAAGGGCAATAC
*PHAVU_006G058400g*	F-AACACCTTGCCACAAGCACTA; R-GCCAGGAAAGAAACACCCATA
*PHAVU_006G058700g*	F-TCAAAGGGTGAAGATGGCAAAT; R-TGGTGGCTCAATATCAGGGTC
*PHAVU_L008300g*	F-CAACTTCTTTGCCATCACTTCC; R-CCTCCCATACTCAAACCCTCAA
*PHAVU_006G029200g*	F-GGAACGAAGTGGTGGAATGG; R-AAGAATGACAAAGTGGGAGGC
*IDE*	F- GCAACCAACCTTTCATCAGC; R -AGAAATGCCTCAACCCTTTG

The expression of 7 *CesA* genes were evaluated and the results showed that these genes in *CesA* subfamily diaplayed temporal variations in different pod development of common bean. The expression of 5 *CesA* genes showed a trend of first increasing in stage S2 and then decreasing in stage S3 (*PHAVU_005G022100g*, *PHAVU_009G094200g*, *PHAVU_002G188600g*, *PHAVU_004G093300g* and *PHAVU_009G205200g*), while the expression of *PHAVU_003G154600g* showed an opposite trend (Fig. 7A). Moreover, the expression level of *PHAVU_007G190300g* decreased with the pod development (Fig. 7A)*.*

In *Csl* genefamily*,* we found that all *Csl* genes selected were expressed at all three stages of pod development. *PHAVU_001G211000g, PHAVU_011G020100g* in *CslD* subfamily, *PHAVU_005G020100g* in *CslB* subfamily showed similar expression pattern: increasing in the S2 stage and decreasing in the S3 stage, which is similar to the expression of *PHAVU_005G022100g*, *PHAVU_009G094200g*, *PHAVU_002G188600g*, *PHAVU_004G093300g and PHAVU_009G205200g* in *CesA* subfamily (Fig. 7B)*. PHAVU_002G136300g* in *CslD* subfamily, *PHAVU_009G2260000g* in *CslG* subfamily and *PHAVU_L008300g* in *CslE* subfamily diaplayed similar expression pattern: decreasing in the S2 stage and slight increasing in the S3 stage. Whereas *PHAVU_008G279800g* and *PHAVU_006G058700g* in *CslE* subfamily showed similar expression trend, but the expression level of *PHAVU_008G279800g* and *PHAVU_006G058700g* in S3 stage significantly higher than the expression level in S1 stage. In addition, the expression level of *PHAVU_005G1163000g* in *CslB* subfamily decreased with the development of pod, the expression level of *PHAVU_011G101500g* in *CslB* subfamily and *PHAVU_003G290600g* in *CslG* subfamily increased only in the S3 stage, the expression level of *PHAVU_003G023000g* in *CslD* subfamily significantly increased in the S2 and S3 stages (Fig. 7B).

## Discussion

Until now, the *CesA/Csl* gene family has been extensively characterized in many plant species, including Arabidopsis, barley, cotton, rice, sorghum, soybean [11, 28–32]. However, this gene family remain unidentified and uncharacterized in common bean. In this study, we conducted a genome-wide survey and identified 39 putative *CesA/Csl* genes in common bean genome (Fig. 1 and Table 1). This results coupled with the sequence alignment, phylogenetic analysis, gene structure construction, chromosome location and expression analysis, could provide important clues in understanding the roles of the *CesA/Csl* superfamily in in pod development in higher plants.

The *CesA/Csl* gene family found across plant species may be subcategorized into nine groups: CslA-CslH and CslJ [2, 33]. All land plants contain CslA, CslC, and CslD, while CslF and CslH are found only in grasses, and cereals do not usually contain CslB or CslG [34, 35]. Using phylogenetic analysis, the 41 *CesA/Csl* genes in *Arabidopsis* were categorized into one CesA group and six Csl groups (Csl A-E and G) [11]. In this study, the phylogenetic analysis showed that the 39 putative *CesA/Csl* genes in common bean could be classified into 5 subfamilies: CesA, CslD, CslB, CslG, and CslE (Fig. 1), consistent with studies of plants and algae [1, 35, 36]. From the phylogeny we can found that CslD is close to CesA, which is consistent with the earlier reports suggesting a common origin and conserved domians of this two families [37].

Among them, 15 putative Cellulose Synthase genes clustered into the CesA gene family, which was the most abundant genes among the 40 *CesA/Csl* genes, while the remaining 24 genes clustered into the other 4 CesA/Csl subfamilies (Fig. 1), suggesting that they have experieced extensive expansion and diversification [33].

Investigation of gene structure and function lends a better understanding of the evolution of a gene family, revealing the divergence, conservation, or expansion of a given gene family [32, 38–40]. Similar to other plants, such as soybean [32] and tomato [31], most *CesA/Csl* genes (*CesA* and *CslD* members) share a similar gene structure in each subfamily (Fig. 2), suggesting that they are highly conserved. In contrast, members in *CslB*, *CslG* and *CslE* subfamily exhibit variable gene structures possibly due to chromosome fusion and/or rearrangement [40]. Therefore, tandem or segmental duplication events in the *CesA/Csl* gene family have resulted in shared exon/intron structures and similar structural organization in each gene subfamily. Phylogenetic and domain analyses confirm these results. Chromosome mapping in this study further revealed that the tandem duplications also existed in *CesA/Csl* gene families (Fig. 5).

The *cis*-elements analysis detected a larger amount of *cis*-elements in the putative promoter regions of the *CesA/Csl* genes in common bean, which suggested that *CesA/Csl* genes might have potential roles in many signaling pathways.

*CesA/Csl* genes have been found to play an important role in plant cell walls in the biosynthesis of cellulose and hemicellulose [7, 16, 41]. During the pod development of common bean, the expression profiles of 21 CesA/Csl genes were revealed by RT-qPCR. The results showed that all 21 CesA/Csl genes were expressed in all three pod development stages, suggesting that all these genes are necessary for the pod growth. Most *CesA* genes in this study expressed highly in the young pod (S2 stage), which is in accordance with the results found in soybean [32], suggesting that these *CesA* genes may be involved in cellulose synthesis during the early pod development stage in common bean. We also found that 3 genes in *CslD* subfamily showed high expression level in the early pod development stage. And the expression level of genes in *CesA* and *CslD* subfamilies is higher than that of other *Csl* genes, which implies that gene members in *CesA* and *CslD* subfamilies are more actively involved in seed development than other *Csl* genes. Therefore, future investigation should aim to identify each *CesA/Csl* gene’s function in common bean.

## Conclusions

Based on the genomic data, 39 cellulose synthase genes were identified from common bean. The genes encoding these proteins were distributed unevenly on 10 chromosomes, and there were 3 tandem duplications. These 39 cellulose synthase proteins could be divided into five subfamilies according to their structure and phylogenetic relationship, members in the same subfamily share conserved exon-intron distribution and motif compositions. Based on the analysis of *cis*-element in the promoter region, we found abundant and distinct *cis*-acting elements, which indicate that the cellulose synthase gene family plays a vital role in the growth and development of common bean. Additionally transcriptional analysis showed that 21 *CesA/Csl* genes selected were constitutively expressed in the pod development, *CesA/Csl* gene members in different groups showed different expression trend at three stages of pod development. In general, this study revealed a putative biochemical and physiological functions of cellulose synthase genes in common bean, which provides a foundation for further function identification of *CesA/Csl* gene family.

## Methods

### Identification of *CesA/Csl* gene family in common bean

The Hidden Markov Model (HMM), which established by 39 CesA/Csl protein sequences of Arabidopsis, was used to search the CesA/Csl gene family in common bean genomes at an e-value cutoff of 1e^−10^. To ensure genes identified with HMM model were accurate, further filtering of unique sequences was performed according to typical structural features of plant CesA/Csl proteins. The Phytozome 11.0 (https://phytozome.gji.doe.gov/) and ExPASy databases (https://web.expasy.org/compute_pi/) were used to obtain gene ID / name, chromosome location, peptide length and isoelectric point/molecular weight, and functinal annotation information [42]. TMHMM v. 2.0 was used to predict the TMDs for each putative peptide (http://www.cbs.dtu.dk/services/TMHMM/).

### Sequence and phylogenetic analyses

ClustalW was used to perform alignments of both CesA/Csl nucleotide and amino acid sequences. Including amino acid sequences of cellulose synthase proteins from Arabidopsis and common bean, the phylogenetic analysis was performed using a neighbor-joining tree method with 1000 bootstrap replicates in the software program MEGA5, which was also used to visualize the phylogenetic analysis. Protein subcellular location were analyzed by WoLF PSORT (http://psort.nibb.ac.jp).

### Gene structure, motif identification and chromosome localization

Gene Structure Display Server v2.0 (http://gsds.cbi.pku.edu.cn/index.php) was used to analyze the exon- intron structure of these genes [43]. MEME program (http:// meme.sdsc.edu/meme/cgi-bin/meme.cgi) was employed to analyze the protein sequences for the confirmation of the motifs. InterProScan (http://www.ebi.ac.uk/Tools/InterProScan/) was used to annotated the motifs.

The chromosome distribution of all cellulose synthase genes of common bean was identified, and the location of CesA/Csl genes was drafted with MapChart v2.0 [44]. Phytozome 11.0 Network database (https://phytozome.jgi.doe.gov/) was also used to obtain genomic DNA and complementary DNA (cDNA) sequences of the putative cellulase synthase genes used in this analysis.

### *Cis*-Element analysis of putative promoter regions and synteny analysis

Using the Phytozome 11.0 network database (https://phytozome.jgi.doe.gov/), 2 Kbp regulatory regions upstream from the start site of translation of CesA/Csl genes were retrieved. Then, the PlantCARE online was used to investigate the putative *cis*-regulatory elements in these promoter region sequences. The location of *cis* elements was annotated and displayed in a figure by building a physical gene map using a Perl and Scalable Vector Graphics (SVG) script. Finally, syntenic relationships of all *CesA/Csl* were analyzed using Circoletto [45].

### Expression analysis of *CesA/Csl* gene family in *Phaseolus vulgaris*

Bean seeds were grown in pots in the open-air soils. Pods were harvested at different stages: 7 (S1), 14 (S2), and 21 (S3) days after flowering. Then, the total RNA of these pods was isolated using the Promega Plant RNA Kit (Promega, Beijing, China) according to the manufacture’s instructions. Single-stranded cDNA was synthesized using 2 μg of total RNA and Oligd(T)18 primer with the Takara RT-PCR system in a total volume of 25 μl according to the protocol. Three independent PCR reactions were carried out for the 63 putative genes using SYBR Green Supermix (Takara) according to the manufacturer’s protocol in an ABI 7500 Real-time system (ABI, CA, USA). IDE (insulin degrading enzyme) was used as an internal control to normalize the expression of CesA/Csl genes according to Borges [46]. Gene-specific DNA primers for qPCR are listed in Table 2.

### Statistical Analysis

Statistical analyses were performed in Excel and SPSS v17.0 (link/cite SPSS.) The significance threshold between samples was *p* < 0.05, and all results of expression data were indicated as averages ±standard deviations (SDs).

## Supplementary Information

**Additional file 1.** Supplementary data associated with this article can be found in the appendix. The information of 20 conserved motifs of Cellulose synthase genes in Phaseolus vulgaris detected by the online tool MEME.

**Additional file 2 **The information of cis-elements in promoter sequences of Cellulose synthase genes in *Phaseolus vulgaris.*

**Additional file 3 **The protein sequences of Cellulose synthase genes in *Phaseolus vulgaris.*

**Additional file 4 **The protein sequences of Cellulose synthase genes in *Glycine max.*

**Additional file 5 **The protein sequences of Cellulose synthase genes in *Arabidopsis thaliana.*

**Additional file 6 **The raw data of characterization of the putative Cellulose Synthase genes in *Phaseolus vulgaris.*

## Data Availability

The datasets used and/or analysed during the current study provided in Supplementary Material File 6.

## Abbreviations

CesA: Cellulose synthase A
Csl: Cellulose synthase-like
